# An in-depth bioinformatic analysis of the novel recombinant lumpy skin disease virus strains: from unique patterns to established lineage

**DOI:** 10.1186/s12864-022-08639-w

**Published:** 2022-05-24

**Authors:** Alena Krotova, Olga Byadovskaya, Irina Shumilova, Antoinette van Schalkwyk, Alexander Sprygin

**Affiliations:** 1 Federal Center for Animal Health, Vladimir, Russia; 2 Agricultural Research Council - Onderstepoort Veterinary Institute, Pretoria, South Africa

**Keywords:** LSDV, Capripoxvirus, Genome, Sequencing, Recombination

## Abstract

**Background:**

Since the first description of lumpy skin disease virus (LSDV) in Africa in the 1920’s, it has brazenly spread beyond Africa into the Middle East, Europe and most recently Asia. In 2017 the first atypical LSDV recombinant strain was reported in Russia, composed of both a live-attenuated Neethling vaccine strain and Kenyan vaccine strain. An increase in LSDV research enabled a public release of numerous full genome sequences of unique recombinant LSDV strains from Kazakhstan, Russia, China and Vietnam. Prior to the recombinant strain first described in China in 2019, every new recombinant strain was genetically unique and each of these recombinants clustered in a monophyletic lineage. In this work, we provide the complete genome sequences of two novel recombinant strains of LSDV from Russia and attempt to gain more insight into genomic composition of all the recombinant strains currently available. This analysis will provide new insight into the global molecular epidemiology of LSDV.

**Results:**

By sequencing and analyzing two novel recombinant strains Khabarovsk/2020 and Tomsk/2020, this study investigates the differences and similarities of all five the available recombinant LSDV lineages from different countries based on the SNPs inherited from the aforementioned parental strains. A total of seven recombinant strains: LSDV/Russia/Saratov/2017, LSDV/Russia/Udmurtya/2019, LSDV/KZ-Kostanay/Kazakhstan/2018, LSDV/Russia/Tyumen/2019, LSDV/GD01/China/2020 Khabarovsk/2020 and Tomsk/2020 were examined. It was observed that strains isolated prior to 2020 were composed of unique combinations of open reading frames, whilst from 2020 onwards all circulating strains in Russia and South-Eastern Asia belonged to a single lineage radiating out in the region. The first representative of this lineage is LSDV/GD01/China/2020. Interestingly, the other four unique recombinant strains as well as the newly established lineage, exhibit consistent patterns of targeted selection pointing to regions constantly selected for during the recombination-driven processes.

**Conclusion:**

This study highlights the inexplicable emergence of novel recombinant strains to be unique introductions of sibling viruses, with the most recent recombinant lineage establishing as the dominant strain across the south eastern Asian countries as evidenced by full genome sequence data. Overall, these findings indicate that LSDVs are subjected to accelerated evolutionary changes due to recombination in the face of homologous live attenuated vaccines as well as the slow genetic drift commonly observed in capripoxviruses curculatign in the field with hardly any genetic changes over decades.

**Supplementary Information:**

The online version contains supplementary material available at 10.1186/s12864-022-08639-w.

## Introduction

Lumpy skin disease virus (LSDV) is a poxvirus in the genus *Capripoxvirus* and the causative agent of lumpy skin disease (LSD). The disease was originally described as pseudo-urticaria in Northern Rhodesia (Zambia) in 1929, but spread rapidly throughout Africa where it circulated for *circa* 80 years. By 2015 the disease spread across the Middle East to Russia, Greece and Serbia [1–3], whilst in 2021 the transboundary disease expansion reached China, India, Bangladesh, Vietnam and Thailand [4–8]. It is evident from the published genomic data, that the circulating strains in Russia, since 2017, were not clonal but belonged to different recombinant lineages of LSDV [9]. In this regard the observed dynamic and transboundary potential of the disease requires urgent efforts to better understand the fundamental aspects of its nature.

Since LSDV was a neglected disease of African countries during the previous century research on it was limited and thus little is known about the evolutionary biology of it. In the last decade, following the LSD insurgence into the northern latitudes, capripoxvirus research enjoyed an intensive and continuous boost [10]. Despite all the new efforts, a major gap in the current knowledge relates to the molecular evolution of LSDV. Before the accessibility of full genome LSDV sequences, Gershon et al. 1989 proposed that all capripoxviruses came into being by recombination from a single ancestor [11]. This remained as a hypothesis until a naturally occurring recombinant strain of LSDV composed of the live attenuated vaccine (LAV) strains Neethling/Vaccine/1959 and a Kenyan KSGPO-like strain was recovered from the field in Saratov, Russia during an active outbreak in 2017 [2]. Since the description of LSDV/Russia/Saratov/2017, additional complete genome sequences of vaccine-like recombinant LSDVs have been made available through GenBank [9]. Novel recombinant viruses of LSDV were reported from Russia, Kazakhstan, the People’s Republic of China and in Vietnam [7, 12]. An interesting feature of these novel variants is their capability to overwinter in climatic winters due to alternative modes of spread in the climatic conditions of the northern latitudes and the ease of travel into and across administrative boundaries [2, 7, 9, 13].

These novel recombinant isolates do not seem identical to each other, but share genetic patterns obtained from either parental strain (Kenyan KSGPO-like and LSDV/LW-1959/Vaccine), resulting in novel phenotypes and enhanced viral fitness [14]. Considering the dearth of data on molecular evolution of capripoxviruses, little can be accomplished in the understanding how and which loci are selected over the course of evolution. Having said this, it is therefore imperative that the likelihood and frequency of generating novel recombinant viruses by nature be determined in order to pinpoint sites targeted by selection. Harnessing these mechanisms would enable an enhanced control of the molecular evolution of capripoxviruses. To our knowledge, unfortunately, such a study is lacking.

In this study, we provide additional full genome sequences of two recombinant LSDVs and gain insights into the conservation of regions selected for by recombination across the available LSDV genomes by comparing full-genome sequences of all the known novel recombinant strains from Russia, Kazakhstan, China and Vietnam with their parental strains from Africa.

## Materials and methods

### Strain

Outbreaks of LSD were reported in the Khabarovskiy Krai and Tomsk Oblast in the Russian Federation, in 2020. Samples of these outbreaks were submitted to the Federal Centre for Animal Health in Vladimir for laboratory confirmation of the disease and two LSDV strains designated LSDV/Russia/Khabarovsk/2020 and LSDV/Russia/Tomsk/2020 were successfully isolated on lamb testis cells as previously described [2]. Viral genomic DNA was extracted and Nextera XT libraries prepared for each of the samples according to manufacturer’s instructions (Illumina, USA). The sequences were determined using a MiSeq Benchtop sequencer (Illumina, USA) according to manufacturer’s instructions as previously described [2]. The complete genome sequences were individually de novo assembled using (~ 222 000 reads) to generate a single sequence representing each of the isolates with an average coverage of (157), in CLC Genomics v9. (www.CLCbio.com). The complete coding sequences of LSDV/Russia/Khabarovsk/2020 and LSDV/Russia/Tomsk/2020 were submitted to GenBank under the accession numbers (OM793603 and OM793602).

### Phylogenetic analysis

An alignment using the newly sequenced LSDV/Russia/Khabarovsk/2020 and LSDV/Russia/Tomsk/2020, along with complete LSDV genomes obtained from GenBank was generated in CLC Genomics. The sequences included in these analyses were: LSDV/KSGPO-240/Kenya (KX683219), LSDV/LW-1959/Vaccine (AF409138); LSDV/Russia/Saratov/2017 (MH646674), LSDV/Russia/Udmurtya/2019 (MT134042), LSDV/KZ-Kostanay/Kazakhstan/2018 (MT992618), LSDV/Russia/Tyumen/2019 (OL542833) and LSDV/GD01/China/2020 (MW355944) (Supplementary Table 1). Sequences from the recent outbreaks in Hong Kong (2020) (MW732649) and Vietnam (2020) (MZ577073, MZ577074, MZ577075 and MZ577076) as well as Taiwan (2020) (OL752713) were included in only certain analysis, since they share a high percentage sequence identity to LSDV/GD01/China/2020 (MW355944). This alignment was used to generate a Maximum likelihood phylogenetic tree using GTR G + I with 1000 bootstrap iterations using MEGA X [15].

### Bioinformatic analysis

Seven individual recombinant analyses were performed in RDPv4 using in each set LSDV/KSGPO-240/Kenya (KX683219) and LSDV/LW-1959/Vaccine (AF409138) as well as one of the novel recombinant strains: LSDV/Russia/Saratov/2017 (MH646674), LSDV/Russia/Udmurtya/2019 (MT134042), LSDV/KZ-Kostanay/Kazakhstan/2018 (MT992618), LSDV/Russia/Tyumen/2019 (OL542833), LSDV/GD01/China/2020 (MW355944) and the newly described isolates from LSDV/Russia/Khabarovsk/2020 and LSDV/Russia/Tomsk/2020 [16]. Each analysis in RDP was performed using a bootscan/rescan recombination test, MAXCHI, GENECONV, CHIMAERA and the SISCAN method with default settings over a 200 bp sliding window as previously described [2, 9]. The predicted recombination events with significant statistical support by three or more methods were displayed on a single graph in Excel.

The same alignment utilized during the phylogenetic analysis was used to identify the nucleotide variants in each of the sequence for the 2184 SNPs previously identified between LSDV/KSGPO-240/Kenya (KX683219) and LSDV/LW-1959/Vaccine (AF409138) using CLC Genomics v9 [17]. The SNPs within each of the novel recombinant isolates were assigned as either being identical to KSGPO-240 (K) or identical to LW-1959 (V) in Excel. The number of SNPs and their corresponding genomic position were counted and subsequently analysed based on their possible role on the functionality of the predicted protein encoded by the affected open reading frame (ORF) they are located in.

## Results

**Fig. 1:**
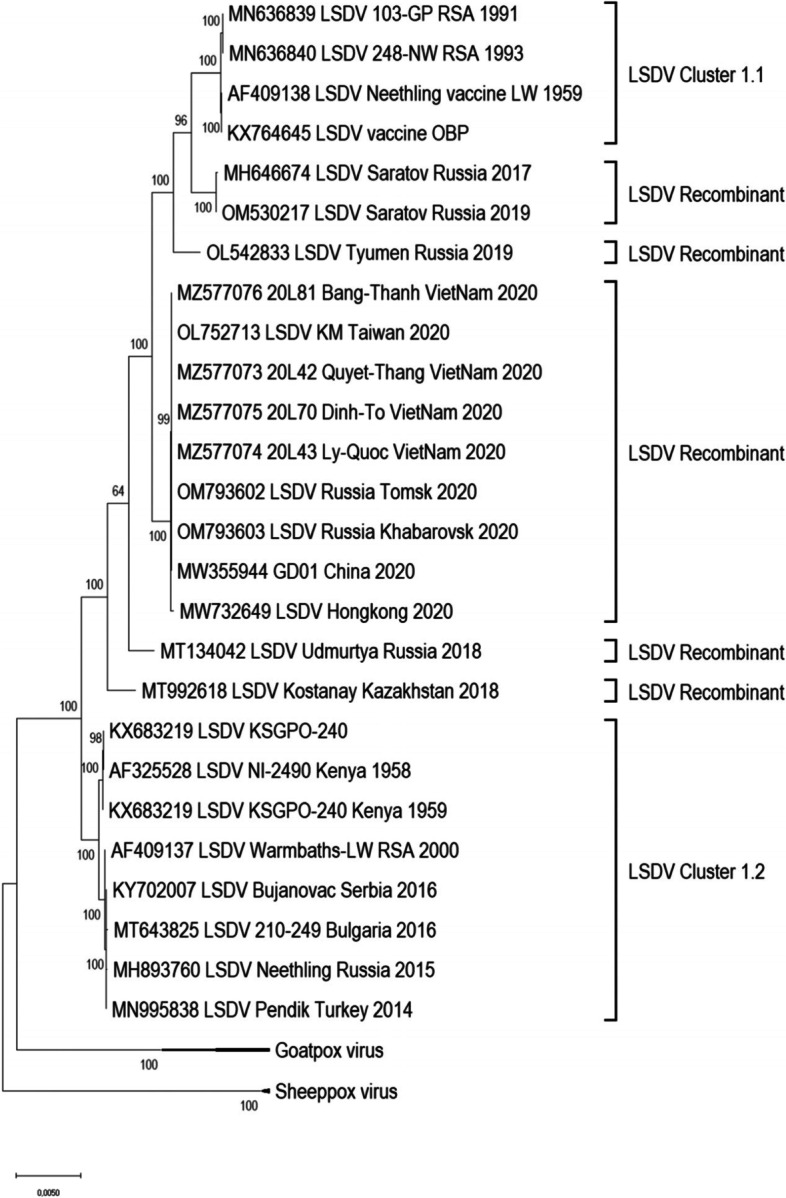
Maximum likelihood phylogenetic tree representing genetic relationship of the complete genome sequences of 26 LSDVs, 2 GPVs and 2 SPPVs

In 2020, possible outbreaks of LSD were reported in the Khabarovsk Krai and Tomsk Oblast of Russia. Samples from cattle with clinical symptoms were aseptically transferred to the Federal Centre for Animal Health in Vladimir for laboratory confirmation of the disease. The samples tested positive using qPCR and viruses, designated LSDV/Russia/Khabarovsk/2020 and LSDV/Russia/Tomsk/2020, were successfully isolated on lamb testis cells. Additional virus characterization of these isolates resulted in the elucidation of the complete genome sequences of LSDV/Russia/Khabarovsk/2020 and LSDV/Russia/Tomsk/2020. The two consensus sequences consisted of 150,222 and 150,209 bp respectively. Pairwise comparisons using the complete genome sequences of LSDV/Russia/Khabarovsk/2020 and LSDV/Russia/Tomsk/2020, together with LSDV sequences available from GenBank, indicated that these viruses share a significantly high (99.99—100%) percentage sequence identity with LSDV/GD01/China/2020. Phylogenetically, they cluster with sequences from outbreaks in China, Taiwan and Vietnam, whilst all the recombinant strains form five monophyletic groups (Fig. 1). The sequence LSDV/GD01/China/2020 from China was used as representative of this Asia/2020 group in subsequent analysis since it represents the first isolate of this cluster.

**Fig. 2:**
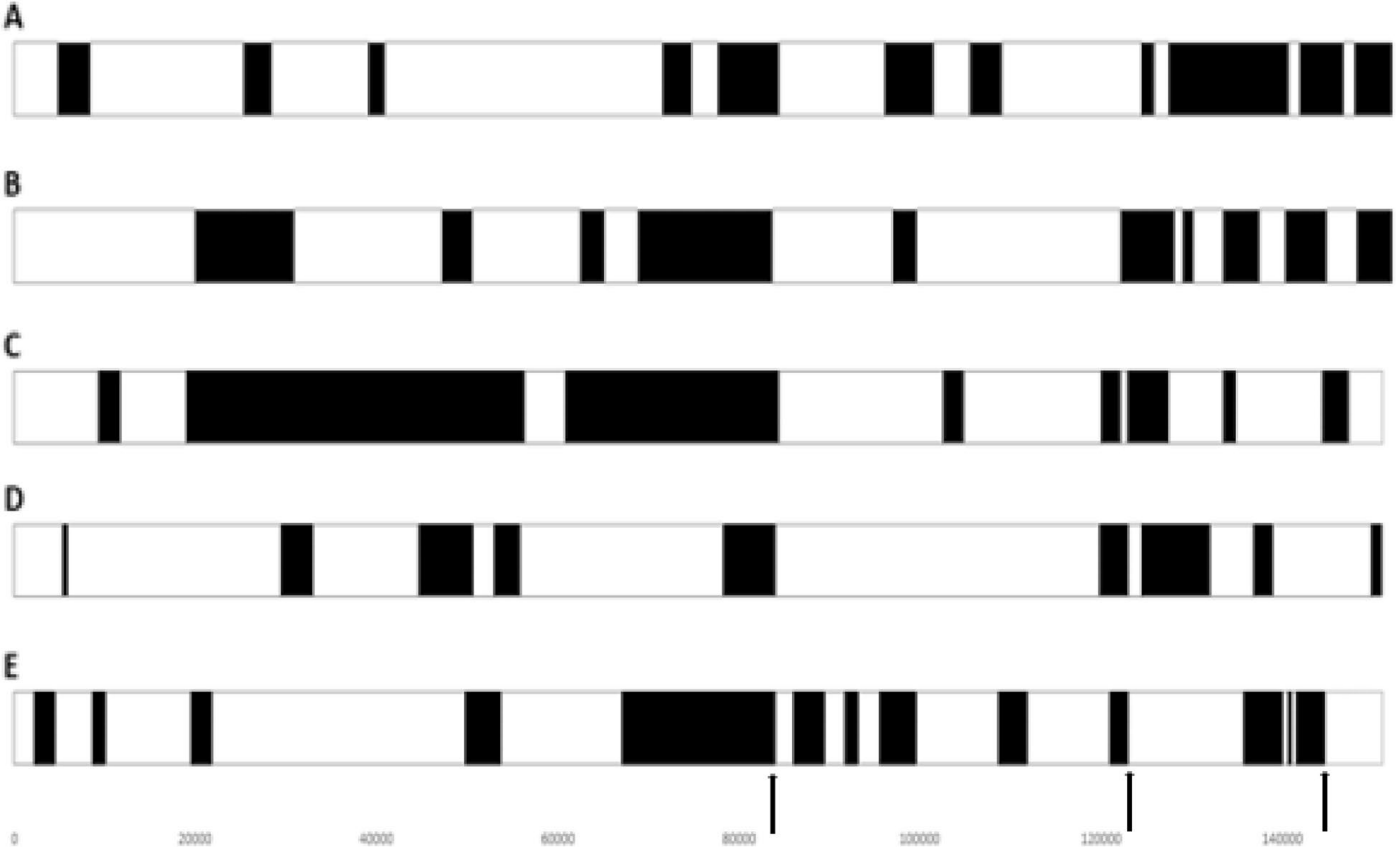
Graphical representation of the position of the predicted recombination events across the five strains with the highest statistical significance. The events are indicated in black in the white backbone of the viral genome. Possible regions selected for by recombination are indicated by arrows. **A** LSDV/KZ-Konstanay/Kazakhstan/2018. **B** LSDV/Russia/Tyumen/2019. **C** LSDV/Russia/Udmurtya/2019. **D** LSDV/Russia/Saratov/2017. **E** LSDV/GD01/China/2020

The two new sequences, LSDV/Russia/Khabarovsk/2020 and LSDV/Russia/Tomsk/2020, were investigated for possible recombination events using RDPv4 and since the predicted recombination events were identical to the LSDV/GD01/China/2020 sequence, it was decided to only include the latter in the subsequent analysis (results not shown). Each of the recombinant strains (LSDV/Russia/Saratov/2017, LSDV/Russia/Udmurtya/2019, LSDV/KZ-Kostanay/Kazakhstan/2018, LSDV/Russia/Tyumen/2019 and LSDV/GD01/China/2020) in combination with the previously identified two parental sequences [2, 9] were individually analysed in RDPv4 and possible recombination events were predicted. The recombination events with significant statistical support by three or more analysis methods are indicated with black boxes across the genome sequences in Fig. 2. Direct comparison of these recombination events in all five recombinant viruses indicated a unique mosaic distribution of patterns for each strain (Fig. 2). Certain of the predicted recombination events occurred in the same genomic regions of two or more strains, while all of the five strains had a recombination event predicted around 80,000 bp. In addition, breakpoint biases were observed at around 84,000, 124,000 and 141,000 bp over the five sequences (Fig. 2).

**Fig. 3:**
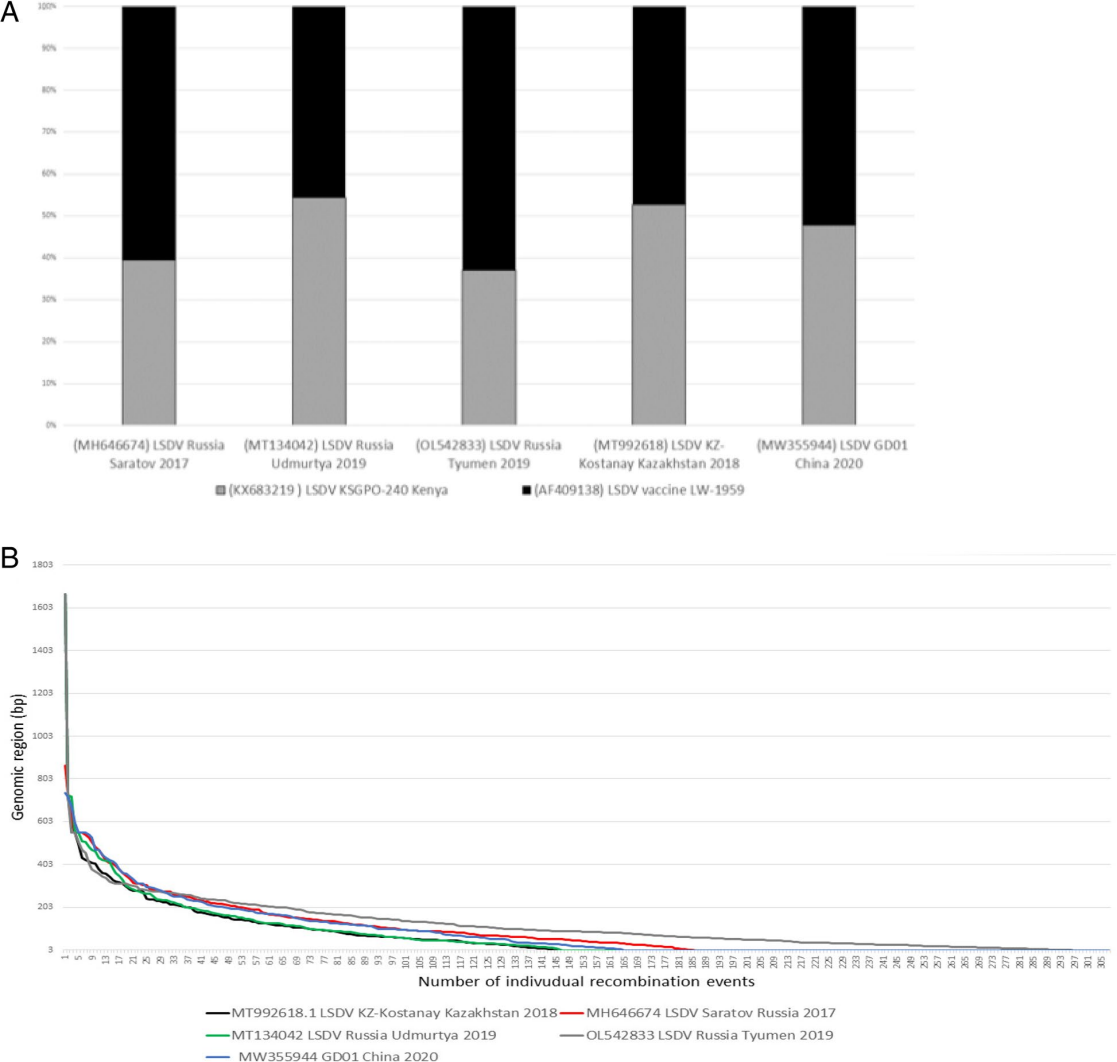
**A** Number of SNPs in LSDV/Russia/Saratov/2017, LSDV/Russia/Udmurtya/2019, LSDV/Russia/Tyumen/2019, LSDV/KZ-Konstanay/Kazakhstan/2018 and LSDV/GD01/China/2020 identical to LSDV/KSGO-2490/Kenya in grey or identical to Vaccine/LW-1959 in black. **B** Conversion track of the calculated recombination events for LSDV/Russia/Saratov/2017 (red), LSDV/Russia/Udmurtya/2019 (green), LSDV/Russia/Tyumen/2019 (grey), LSDV/KZ-Konstanay/Kazakhstan/2018 (black) and LSDV/GD01/China/2020 (blue)

Since the predicted parental strains of all of these recombinant viruses were a Kenyan KSGPO-like and LSDV/Vaccine/LW-1959-like strain, it was imperative to investigate the genetic relationship between each of the five recombinant strains in relation to the two parental sequences and identify the regions selected for by recombination [2, 9]. Previously, 2184 SNPs have been identified between LSDV/Ni-2490/Kenya/1958 (AF325528) and LSDV/Vaccine/LW-1959 and only 2—4 SNPs between LSDV/Ni-2490/Kenya/1958 and LSDV/KSGPO-240/Kenya as well as LSDV/Vaccine/LW-1959 and its derivative commercially available vaccines [17, 18]. Of the 2184 SNPs between LSDV/KSGPO-240/Kenya and LSDV/Vaccine/LW-1958, 328 are located in intergenic regions (IGR), while the remaining 1856 located in ORFs could be classified as 1258 synonymous SNPs and 588 non-synonymous. Using the complete genomes of the five recombinant viruses, each of the 2184 SNPs were assigned to be either identical to LSDV/KSGPO-240/Kenya (K) or LSDV/LW-1959/Vaccine (V). LSDV/Russia/Saratov/2017, LSDV/Russia/Tyumen/2019 and LSDV/GD01/China/2020 had respectively 60,4%, 63% and 52% of the SNPs identical to LSDV/Vaccine/LW-1958, whilst LSDV/Russia/Udmurtya/2019 and LSDV/KZ-Kostanay/Kazakhstan/2018 had 54% and 52,7% identical SNPs to LSDV/KSGPO-240/Kenya (Fig. 3A). This confirmed the phylogenetic relationship observed using the Maximum likelihood phylogenetic tree, that the five recombinant strains are not identical as well as their positions in the phylogenetic tree in regard to either cluster 1.1 or 1.2 (Fig. 1). The recombination frequencies were determined for each of the five strains, by defining a recombination event when a SNP identical to the one parent was downstream of a SNP identical to the other parent [19]. The calculated average number of crossover events was 196 when analysing the 2184 SNPs over the five recombinant sequences. This implies that over a ~ 151 kb genome, there was a calculated 1 SNP every 69 bp and this translates to an average conversion tract of 150 bp (Fig. 3B).

**Fig. 4:**
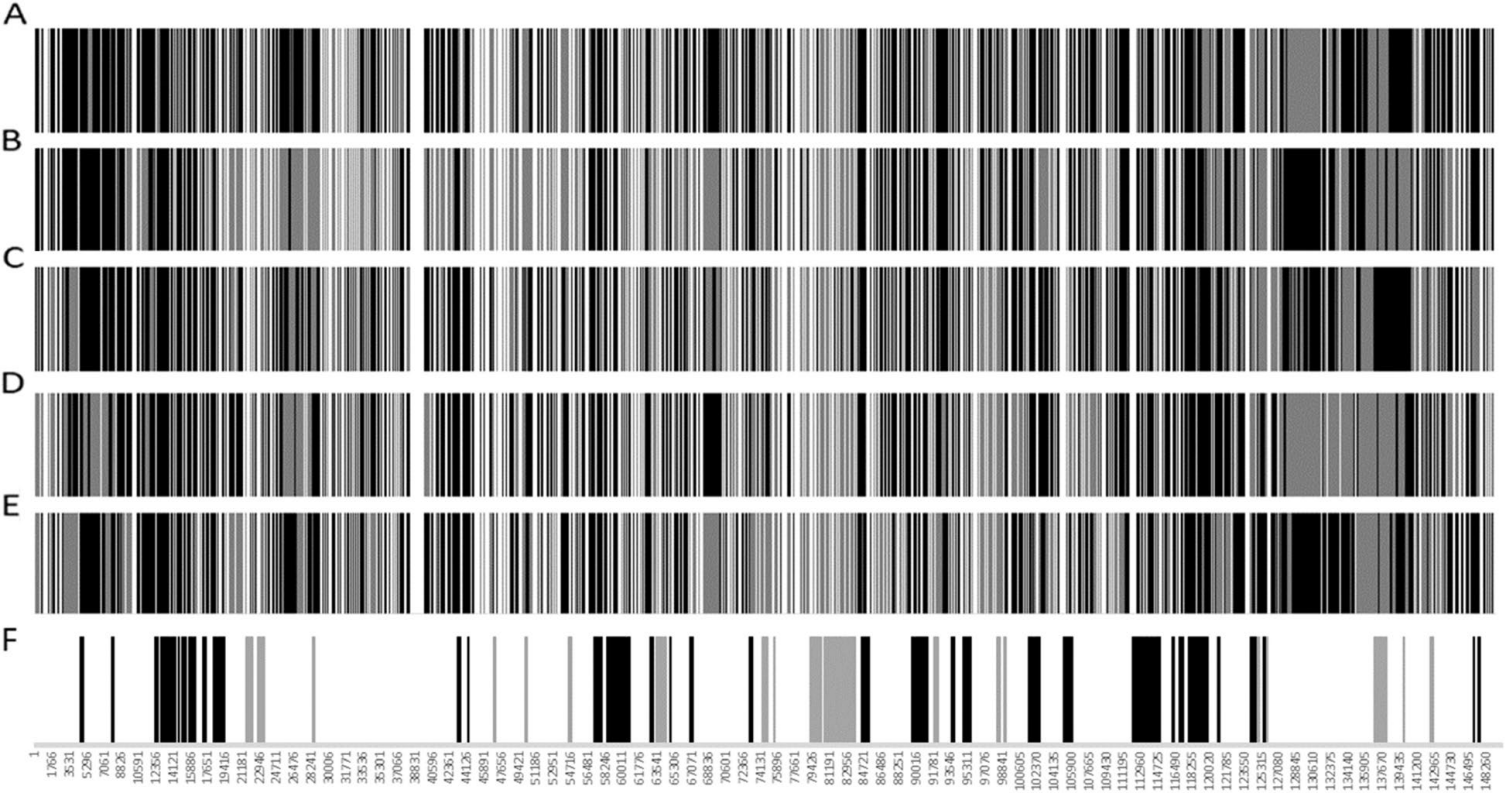
Graphical representation of the position of each of the SNPs in the genomes of LSDV/Russia/Saratov/2017 (**A**), LSDV/Russia/Udmurtya/2019 (**B**), LSDV/Russia/Tyumen/2019 (**C**), LSDV/KZ-Konstanay/Kazakhstan/2018 (**D**) and LSDV/GD01/China/2020 (**E**). Positions identical in all the recombinant strains (**F**). SNPs identical to LSDV/KSGO-2490 were indicated in grey or in black when identical to Vaccine/LW-1959

A graphical representation of the 2184 SNPs within each of the five recombinant strains, in relation to the two parental strains is presented in (Fig. 4). If a SNP in one of the five recombinant strains was identical to LSDV/KSGPO-240/Kenya it is indicated in grey, while SNPs identical to LSDV/Vaccine/LW-1958 are represented with black lines. The clustering patterns of the five recombinant strains, indicates that they are not identical, but siblings or independently created from recombination events between the two parental strains, since all the SNPs could be assigned to either parent (Fig. 4A-E). Even though the five recombinant strains are unique, 306 SNPs were identical between all of them. Of these, 113 were identical to LSDV/KSGPO-240/Kenya and 193 identical to LSDV/Vaccine/LW-1959. These SNPs are clustered into 63 ORFs with 198 synonymous and 86 non-synonymous SNPs, while 22 were in IGRs (Fig. 4F). Interestingly, the SNPs form clusters within neighbouring ORFs in order to select for either one of the parental sequences (Fig. 4F). The majority of the ORFs under selection are involved in either transcription, replication or virion formation (Table-S2). The position of amino acid exchanges as well as the function of each of the predicted proteins are indicated in Table-S2.

## Discussion

With the onset of next generation sequencing (NGS) and third generation sequencing technologies (TGS), coupled to the rapid expansion of LSD incursions into the northern latitudes, the number of full length LSDV genomes have increased rapidly over the last decade [2, 20–25]. The availability of recent and archived strains enabled an investigation into the molecular clocks of LSDV evolution, suggesting the estimated divergence time between the Capripox viruses around ~ 19,000 and the common ancestor of both LSDV clusters 1.1 and 1.2 existed ~ 550 years ago [26, 27]. Prior to 2017, the LSDVs could be grouped into two clusters [27–29], but novel LSDV strains isolated from the Russian Federation, Kazakhstan, China and Vietnam clustered well outside these well-established groups (Fig. 1). As previously described, this unique phylogenetic clustering is indicative of recombination events occurring between LSDV strains belonging to both of the major clusters (Clusters 1.1 and 1.2), resulting in novel genetic variants [2, 12, 23]. In this study we contribute complete genome sequences of two additional recombinant LSDV strains, isolated during outbreaks in 2020 in Khabarovsk and Tomsk, in Siberia and the Far East of Russia. These strains share significant sequence homology with viruses isolated from Hong Kong, Taiwan and Vietnam in 2020 as well as an isolate from mainland China in 2020. This is in disagreement with the past data from Russia that indicate that all recombinant LSDV from 2017 to 2019 were unique [2, 9]. The two new strains from Russia in 2020, had identical recombination events predicted, when analyzed using RDPv4, compared to the initial isolate in this group LSDV/GD01/China/2020 (results not shown). This suggests that these viruses could be seen as a clonal continuation of the outbreak dating back to 2019 [6], which spread across multiple national borders of the southeast of Asia, rather than new introductions as was the case for Russia in 2017–2019. These molecular findings mirror the clonal expansion of the classical field strain before 2016 in Africa, the Middle East, Europe and Russia [2, 21]. This particular lineage represented by LSDV/GD01/China/2020 has been established as the dominant strain in the region, due to yet unidentified fitness features. In contrast to the other four recombinant viruses, the viruses from China and Vietnam have an almost equal distribution of SNPs identical to both parental strains (Fig. 3).

The origin of this plethora of novel recombinant strains remains unanswered. The parental viruses involved in the multiple recombination events have been identified as a LSDV/KSGPO-like (either LSDV/Ni-249/Kenya/1958 or LSDV/KSGPO-240/Kenya) and LSDV/Vaccine/LW-1959, or one of its commercially available derivatives [2, 9, 12, 30]. The newly described sequence from Russia/Tyumen/2019 as well as the first recombinant virus, Russia/Saratov/2017, seems to have a LSDV/Vaccine/LW-1959 backbone, with novel exchanges with LSDV/KSGPO-240/Kenya distributed throughout (Fig. 3). The opposite was observed for Russia/Udmurtya/2019 and LSDV/KZ-Kostanay/Kazakhstan/2018 (Fig. 1 and 3A). Despite the differences in the genetic backbone and novel recombination patterns of each novel strain, specific breakpoint hotspots were identified across multiple sequences. These breakpoint hotspots will be subjected to future investigations, but their locations might be incorrectly assigned since they are based on the same set of variable nucleotide positions (VNP), i.e. the ~ 2,100 SNPs observed between the parental sequences [31]. These SNPs and their distribution within each of the five recombinant strains were used to calculate the average number of crossover events at 196 and subsequently the average conversion tract to be 150 bp in size (Fig. 3B). When these calculations were compared to the experimentally induced recombination experiments involving the Vaccinia virus (VACV) clones TP05 and DPP17, where a SNP occurred on average every 140 bp and the average conversion track was 12 kb in length, it is evident that the novel recombinant strains were subjected to a high passage history with a high multiplicity of infection (MOI) [19].

It is hypothesized that the novel recombinant strains were naturally generated to gain a phenotypic advantage or viral fitness in the dramatically different climatic conditions of the northern latitudes where LSDV had never occurred before [9, 32, 33]. This still awaits experimental confirmation. Yet, recently partial sequences of a LSDV isolate from a vaccinated cow from Kenya in 2011 was described, clustering with the same recombination parents as the five recombinant groups described in this study [34]. Comparisons between the partial sequences obtained from the Kenyan/2011 isolate with LSDV/Russia/Saratov/2017 indicated that they were not identical, but similar to the five recombinant virus sequences analysed here a novel recombinant strain [34]. The parental sequences predicted to be involved in the generation of the novel recombinants observed in Kenya, Kazakhstan, Russia and China and Vietnam, are mirrored in the mixed composition recently described in the Lumpivax vaccine batch from Kevevapi, that was extensively used in Kazakhstan since 2016 [10, 35]. Although there is no evidence to identify the time when, and if, the recombinants emerged in the vaccine batch at the manufacturer’s site, evidence of a 2016 batch of Lumpivax vaccine from Kevevapi already displayed both vaccine and field signatures, i.e. contamination already existed (Sprygin A, unpublished findings). These findings clearly demonstrate that the circulation of novel recombinant isolates with divergent recombination patterns cannot be a continuous wave-like spread from outbreaks in the past, but suggest repeated transboundary incursions. Two exceptions to this observation include the reoccurrence of the original recombinant strain isolated in Saratov in 2017, two years later in 2019, indicating that these viruses are capable of overwintering in the climatic conditions of the northern latitudes [13]. The second exception is the vast transboundary radiation of the strain originally described in China/2020 mentioned previously that is seemingly spreading towards the west again.

The analysis undertaken here provides evidence for selection in genomic sites targeted by recombination. Although the recombination events produced genetically unique strains, this study identifies a definite selection bias for specific genomic regions of one parent instead of another (Table-S2). The open reading frames selected for, encodes proteins involved in virus structure (LW008, LW028, LW081, LW095, LW103 and LW122), transcription and DNA replication (LW020, LW049, LW071, LW075, LW084, LW085, LW086, LW087, LW088, LW098, LW110, LW112 and LW119). Multiple neighbouring ORFs were preferentially selected for, which could either be because of the size of the predicted recombination events by RDP, or indicate functional dependency of the resulting proteins in association with each other. The non-synonymous SNPs resulted in the predicted exchange of neutral to charged amino acids and the subsequent increase in the predicted antigenicity profile of the predicted receptor-like proteins. The influence of each of the selected substitutions on the phenotype of the virus should be determined independently.

Based on the mosaic nature of these novel recombinant viruses, the importance of performing whole genome sequencing (WGS) on outbreak samples is reiterated [12]. None of the current PCR assays, capable of differentiating vaccinated from infected animals (DIVA) could identify these recombinant viruses as such, when performed individually [20, 36–39].

In conclusion, five independent individuals, or groups of, novel recombinant LSDVs have been isolated in recent years. Each of these novel recombinant viruses were originally observed in Russia bordering on Kazakhstan where the contaminated vaccine was administered with up to 100% coverage of the cattle population [10]. Subsequently, recombinant strains occurred in China, Vietnam, Russia and other countries of the region. Thus far, only one lineage originally described in China in 2020 seems to have spread to neighbouring countries both south- and northwardly [12], pointing towards the effective preventative properties of the currently available heterologous and homologous vaccines used in affected or countries at risk [24]. Although, the scope of this work only covers LSDV strains sequenced up to 2020, cases of LSD have been reported in Malaysia, Singapore, Thailand and Indonesia in 2021–2022 that are not considered in the analysis due to the unavailability of full genome sequences. Incorporation of new data in future studies will greatly improve our understanding of the global LSD epidemiology and capture important details of LSDV molecular evolution. The identification and analysis of all the novel recombinant strains, again reiterate the limitations of the current DIVA assays, the importance of next generation sequencing of circulating isolates as well as the quality inspections during vaccine productions [35].

## Supplementary Information

**Additional file 1: Table S1.** Information on the sequences used within this study.

**Additional file 2: Table S2.** Non-synonymous SNPs observed in all five the recombinant strains identical to LW-1959 on the left and KSGPO-240/Kenya/1958 on the right.

## Data Availability

The data used to support the findings of the manuscript are included within the article and are available in the public domain at GenBank (NCBI). The complete coding sequences of LSDV/Russia/Khabarovsk/2020 and LSDV/Russia/Tomsk/2020 reported in the paper were submitted to GenBank under the accession numbers OM793603 and OM793602, respectively.
