# Supplementary material for: An in-depth bioinformatic analysis of the novel recombinant lumpy skin disease virus strains: from unique patterns to established lineage
Source: BMC Genomics. 2022 May 24;23:396. doi: 10.1186/s12864-022-08639-w (PMC9131581; doi:10.1186/s12864-022-08639-w)
Supplement: Supplementary file 1 — Additional file 1: Table S1. Information on the sequences used within this study. [file 12864_2022_8639_MOESM1_ESM.docx]

Supplementary Table 1. Information on the sequences used within this study.

| LSDV | Genbank accession number | Country of origin | Year of isolation | Phylogenetic cluster |
| --- | --- | --- | --- | --- |
| LSDV/Ni-2490/Kenya/1958 | AF325528 | Kenya | 1958 | 1.2 (Two SNPs with LSDV/KSGPO-240/Kenya |
| LSDV/KSGPO-240/Kenya | (KX683219) | Kenya | Commercial LAV generated from Kenyan sheep and goat pox viruses isolated from ovines | 1.2 |
| LSDV/LW-1959/Vaccine | (AF409138) | South Africa | LAV released in 1960, based on Neethling type-strain | 1.1 |
| LSDV/Russia/Saratov/2017 | (MH646674) | Russia | 2017 | Novel recombinant |
| LSDV/Russia/Udmurtya/2019 | (MT134042) | Russia | 2019 | Novel recombinant |
| LSDV/KZ-Kostanay/Kazakhstan/2018 | (MT992618) | Kazakhstan | 2018 | Novel recombinant |
| LSDV/Russia/Tyumen/2019 | (OL542833) | Russia | 2019 | Novel recombinant |
| LSDV/GD01/China/2020 | (MW355944) | China | 2020 | Novel recombinant |
| Hong Kong (2020) | MW732649 | China | 2020 | Same cluster as GD01/China/2020 |
| Vietnam (2020) | MZ577073 |  |  | Same cluster as GD01/China/2020 |
|  | MZ577074, |  |  | Same cluster as GD01/China/2020 |
|  | MZ577075 |  |  | Same cluster as GD01/China/2020 |
|  | MZ577076 |  |  | Same cluster as GD01/China/2020 |
| Taiwan (2020) | OL752713 | Taiwan | 2020 | Same cluster as GD01/China/2020 |
| LSDV/Russia/Khabarovsk/2020 | OM793603 | Russia | 2020 | Same cluster as GD01/China/2020 |
| LSDV/Russia/Tomsk/2020 | OM793602 | Russia | 2020 | Same cluster as GD01/China/2020 |
