# Supplementary material for: An in-depth bioinformatic analysis of the novel recombinant lumpy skin disease virus strains: from unique patterns to established lineage
Source: BMC Genomics. 2022 May 24;23:396. doi: 10.1186/s12864-022-08639-w (PMC9131581; doi:10.1186/s12864-022-08639-w)
Supplement: Supplementary file 2 — Additional file 2: Table S2. Non-synonymous SNPs observed in all five the recombinant strains identical to LW-1959 on the left and KSGPO-240/Kenya/1958 on the right. [file 12864_2022_8639_MOESM2_ESM.docx]

**Supplementary Table 2. Non-synonymous SNPs observed in all five the recombinant strains identical to LW-1959 on the left and KSGPO-240/Kenya/1958 on the right. The ORF as well as the predicted function of the corresponding protein according to Tulman et al., is listed. Amino acid exchanges are indicated based on the predicted amino acid of the recombinants on the left, position in the predicted ORF and amino acid of the alternative parental strain on the right. ORF LW098 it is indicated in bold, since it contains selected sites unique to both parents.**

| LSDV/LW-1959/Vaccine | | | LSDV/KSGPO-240/Kenya/1958 | | |
| --- | --- | --- | --- | --- | --- |
| ORF | Predicted protein | Amino acid exchange | ORF | Predicted protein | Amino acid exchange |
| LW008 | Soluble interferon-gamma receptor like protein | D 214 N | LW028 | Palmitylated EEV membrane protein | A 135 T |
| LW019 | Kelch like protein | S 94 A  Stop 124  A 176 T  A 205 S  Q 220 R  N 278 D | LW030 | Pox_F16 superfamily | H 34 N |
| LW020 | Ribonucleotide reductase, small subunit | N 52 S  D 219 E  V 250 D | LW056 | Hypothetical protein | K 171 R  N 174 D |
| LW021 | Hypothetical protein | H 2 R | LW081 | Virion core protein | S 227 N |
| LW022 | Hypothetical protein | N 55 D | LW084 | 70kDa small subunit of early gene transcription factor | N 581 D |
| LW024 | Disulfide bond formation pathway protein | F 123 L | LW085 | DNA-dependent RNA polymerase subunit | M 136 T |
| LW026 | Poxvirus F11 superfamily; Rho signal inhibitor | M 62 I  E 189 K  E 225 K | LW086 | MutT motif; mRNA decapping catalysis | L 191 F  Reading frame: 207 |
| LW049 | RNA-helicase | E 32 D | LW087 | MutT motif; mRNA decapping catalysis | D 12 G  V 46 I  Reading frame: 199 |
| LW071 | DNA-dependent RNA polymerase subunit | H 112 Y | LW088 | ATPase, nucleoside triphosphate phosphohydrolase-I | V 24 I |
| LW075 | RNA polymerase associated protein | D 519 E | **LW098** | **82kDa large subunit of early gene transcription factor** | **T 652 I** |
| LW090 | Rifampicin target | S 277 T | LW102 | Stabilizes membranes during virus assembly | T 162 A |
| LW095 | Putative virion core protein | A 129 S | LW103 | Core protein | P 72 T  S 89 G |
| **LW098** | **82kDa large subunit of early gene transcription factor** | **S 75 N** | LW144 | Kelch and BTB containing protein | I 218 D  S 234 N  Reading Frame: 252  -338 E  Y 375 H  P 385 S |
| LW109 | IMV membrane protein | V 181 F |  |  |  |
| LW110 | Helicase, ATP-dependent transcription termination | R 128 K |  |  |  |
| LW112 | DNA polymerase processivity factor | S 347 R |  |  |  |
| LW119 | DNA-dependent RNA polymerase | Q 85 - |  |  |  |
| LW122 | EEV membrane phosphoglycoprotein | T 56 A |  |  |  |
| LW123 | EEV glycoprotein | S 45 N |  |  |  |
| LW128 | CD47-like putative membrane protein | P 27 S  I 43 V  D 48 N  -53 V  G 57 E  T 80 K  K 157 - |  |  |  |
| LW129 | Similar to myxoma virus virulence factor | D 62 E  T 64 S  V 87 D  D 88 N |  |  |  |
| LW133 | DNA ligase | L 36 P |  |  |  |
| LW151 | Kelch like protein | K 375 T |  |  |  |
